# New Insights Optimize Landing Strategies to Reduce Lower Limb Injury Risk

**DOI:** 10.34133/cbsystems.0126

**Published:** 2024-05-22

**Authors:** Datao Xu, Huiyu Zhou, Wenjing Quan, Xin Ma, Teo-Ee Chon, Justin Fernandez, Fekete Gusztav, András Kovács, Julien S. Baker, Yaodong Gu

**Affiliations:** ^1^ Faculty of Sports Science, Ningbo University, Ningbo, China.; ^2^ Department of Orthopedics, Huashan Hospital, Fudan University, Shanghai, China.; ^3^ School of Chemical and Biomedical Engineering, Nanyang Technological University, Singapore 639798, Singapore.; ^4^ Auckland Bioengineering Institute, University of Auckland, Auckland, New Zealand.; ^5^ Department of Engineering Science, University of Auckland, Auckland, New Zealand.; ^6^ Department of Material Science and Technology, Audi Hungaria Faculty of Automotive Engineering, Széchenyi István University, Gyor, Hungary.; ^7^ Faculty of Engineering, University of Pannonia, Veszprém, Hungary.; ^8^ Department of Sport and Physical Education, Hong Kong Baptist University, Hong Kong, China.

## Abstract

Single-leg landing (SL) is often associated with a high injury risk, especially anterior cruciate ligament (ACL) injuries and lateral ankle sprain. This work investigates the relationship between ankle motion patterns (ankle initial contact angle [AICA] and ankle range of motion [AROM]) and the lower limb injury risk during SL, and proposes an optimized landing strategy that can reduce the injury risk. To more realistically revert and simulate the ACL injury mechanics, we developed a knee musculoskeletal model that reverts the ACL ligament to a nonlinear short-term viscoelastic mechanical mechanism (strain rate-dependent) generated by the dense connective tissue as a function of strain. Sixty healthy male subjects were recruited to collect biomechanics data during SL. The correlation analysis was conducted to explore the relationship between AICA, AROM, and peak vertical ground reaction force (PVGRF), joint total energy dissipation (TED), peak ankle knee hip sagittal moment, peak ankle inversion angle (PAIA), and peak ACL force (PAF). AICA exhibits a negative correlation with PVGRF (*r* = −0.591) and PAF (*r* = −0.554), and a positive correlation with TED (*r* = 0.490) and PAIA (*r* = 0.502). AROM exhibits a positive correlation with TED (*r* = 0.687) and PAIA (*r* = 0.600). The results suggested that the appropriate increases in AICA (30° to 40°) and AROM (50° to 70°) may reduce the lower limb injury risk. This study has the potential to offer novel perspectives on the optimized application of landing strategies, thus giving the crucial theoretical basis for decreasing injury risk.

## Introduction

Landing is an essential sports technique, and its use is particularly frequent in most ball sports [1–5]. The supporting styles for landing can be divided into single-leg and double-leg supports. Compared to double-leg landing support, the use of single-leg landing (SL) support facilitates rapid deceleration and swift changes in direction after landing, so it is used more frequently in exercises [1,5–7]. Different landing patterns can cause different loading effects on the musculoskeletal and soft tissues of the lower limb joints [1,2,7–9]. Lower limb musculoskeletal injuries are also common in different landing patterns, such as anterior cruciate ligament (ACL) injuries, ankle sprains, and patellar tendonitis [1,4,5,7,10,11].

During landing, as the key joint connecting the ankle and hip, the knee has the highest risk of injury [8,12,13]. The most common is the non-contact ACL injury, with more than 80% of its injuries occurring during landing tasks [1,5,6,14]. As one of the main ligaments around the knee joint, the ACL is crucial for maintaining stability in the knee joint [15,16]. After an ACL rupture, the patients experience a general decrease in their exercise level and quality of life. Even after prompt treatment, only 55% of patients return to their daily competitive sport level within the first year [17]. More seriously, ACL ruptures also frequently lead to other pathologic changes, such as osteoarthritis of the knee [14,18–21].

It is also true that ankle sprains, on the other hand, are one of the most common injuries associated with landing, and more than 80% of ankle sprains occur in the lateral [22,23]. Lateral ankle sprain (LAS) causes structural damage to the lateral collateral ligaments (anterior tibiofibular ligament and calcaneofibular ligament) of the ankle joint [23,24]. These structural injuries may cause joint pain, swelling, and related dysfunction. In severe cases, they can also cause re-sprains of the ankle joint and progress to chronic ankle instability [22,24]. An increased ankle inversion angle is recognized as one of the important biomechanical factors causing LAS [23,24]. Ankle inversion is the inward rotation of the ankle joint that turns the sole toward the midline of the body. When the ankle is in greater plantarflexion and inversion, it is considered to be at greater risk for LAS [24–26].

During the landing process, the lower limbs undergo a transfer of load impact pattern from the distal to the proximal: foot-ankle to knee and knee to hip [4,5,13]. The ankle joint and its surrounding muscles and tissues have a crucial function in absorbing the load impact during landing, serving as the initial point for the transfer of this impact [5]. Research has shown that they can withstand impact forces ranging from 2 to 5 times the individual's body weight during an SL [5,9,27,28]. Previous studies have suggested that increasing the ankle initial contact angle (AICA) during landing may increase ankle energy dissipation [5,29], and a greater ankle range of motion (AROM) has also been thought to increase the time to the peak point of vertical ground reaction force (VGRF) [1,3], thereby reducing the impact on the lower limb [5]. However, whether ankle joint motion patterns (AICA and AROM) during SL affect joint energy dissipation and the degree of shock load cushioning across the lower limb, or even if there is some association with lower limb injury, remains to be further explored. It is undeniable that AICA and AROM during SL can be adjusted to a large extent by human autonomic awareness. Consequently, guiding individuals to consciously adjust their ankle movement patterns during landing based on the discovered laws is anticipated to decrease the incidence of lower limb injuries [5,7].

In assessing the ACL injury mechanism, traditional models mainly set ligaments as having linear force–length characteristics and consider them independent of strain rate [30–32]. However, ligaments, as dense connective tissue (DCT), are characterized by short-term viscoelastic strain, which affects the force–length characteristics [31,33]. Hence, to more realistically revert and simulate the ACL injury mechanics, this study developed a knee musculoskeletal model that reverts the ACL ligament to a nonlinear short-term viscoelastic mechanical mechanism (strain rate-dependent) generated by the DCT as a function of strain. By implementing the structural constitutive model, this study calculated the compressive stress on the ligament at different strains [33,34].

Therefore, this study aims to explore an optimized landing strategy to reduce the injury risk of the lower limb. We hypothesized that the variations in ankle motion patterns (AICA and AROM) during SL would be associated with the injury risk of the lower limb. Among these, by appropriately increasing AICA and AROM during SL, the overall injury risk of the lower limb, particularly ACL injury, can be minimized, but this increases the LAS risk. Furthermore, there is an “optimal” ankle movement pattern that can balance the association between the LAS and ACL injury risk, allowing for minimization of the overall injury risk.

During landing, when the body undergoes a greater VGRF, the impact on the joints of the lower limbs is greater [35,36]. Among them, the degree to which the joints and soft tissues of the lower extremities are subjected to loads increases if the landing impact is not effectively dissipated [2,5]. To be exact, insufficient energy dissipation in the lower extremity joints will increase the injury risk [5]. The moment is the torsional effect that the forces around the joint produce, and a larger moment implies greater stress on the muscles and ligaments surrounding the joint [1,26]. These changes in biomechanical outcomes are thought to lead to an increased risk of lower limb injury [26,36,37]. Furthermore, the greater the loading force on the ACL, the greater the risk of ACL tears and strains [5,37].

Therefore, the following works are specified: (a) The peak VGRF (PVGRF), total energy dissipation (TED), peak ankle sagittal moment, peak knee sagittal moment, and peak hip sagittal moment were used to assess the overall injury risk of the lower limb, and explored their relationships with AICA and AROM. (b) The peak ankle inversion angle (PAIA) and peak ankle inversion moment (PAIM) were used to assess the LAS risk, and hypothesized the positive correlation between them and AICA and AROM. (c) The ACL model was developed and constructed to calculate ACL dynamic loading forces, and then the peak ACL force (PAF) was used to assess the ACL injury risk, and hypothesized the negative correlation between them and AICA and AROM. (d) The feasible ankle motion patterns were explored to balance the LAS and ACL injury risk based on the interaction between PAIA and PAF.

## Materials and Methods

### Subjects

Sixty healthy male subjects were recruited for this experiment, and their anthropometric parameters are as follows: age, 22.43 ± 5.02 years; height, 1.84 ± 0.12 m; and body mass, 81.93 ± 12.81 kg. Subjects were screened based on (a) no history of serious lower limb surgery within the past 6 months; (b) no other injury variables that would affect the study; and (c) no other problems that would affect sports performance. We informed the subjects in advance of the experiment’s purpose, requirements and procedures, and they signed the written informed consent form. The Ethics Committee of Ningbo University approved the study protocol (approval number: RAGH20210120).

### Experimental protocol and procedures

Experiments were conducted in the biomechanics laboratory of Ningbo University. The instrument has a motion capture device with an 8-camera system (Vicon Metrics Ltd., UK), a wireless electromyographic (EMG) system with 16 channels (Delsys, Boston, MA, USA), and 2 force plates (AMTI, Watertown, USA), and the sampling frequencies were set as 200 Hz, 1000 Hz, and 1000 Hz, respectively. The current study carried out a lower limb musculoskeletal modeling based on the pipeline constructed from previous models [5,30,31,38]. This musculoskeletal model was mainly constructed and developed based on the 2392 generic musculoskeletal model [31,39]. The current model contains a total of 5 main joints of the lower extremity (hip, knee, ankle, subtalar, and metatarsophalangeal joints), 10 rigid bodies, and 90 musculotendon actuators. Among them, the hip, knee, and ankle joints have 3 degrees of freedom (DOF), and the subtalar joint and metatarsophalangeal joint only have sagittal plane DOF. For the 90 musculotendon actuators, based on the 92 musculotendon actuators of the 2392 model, 6 musculotendon actuators near the spine of the upper limbs were removed, and 4 ACL musculotendon actuators from the left and right knees were added.

**Fig. 1.**
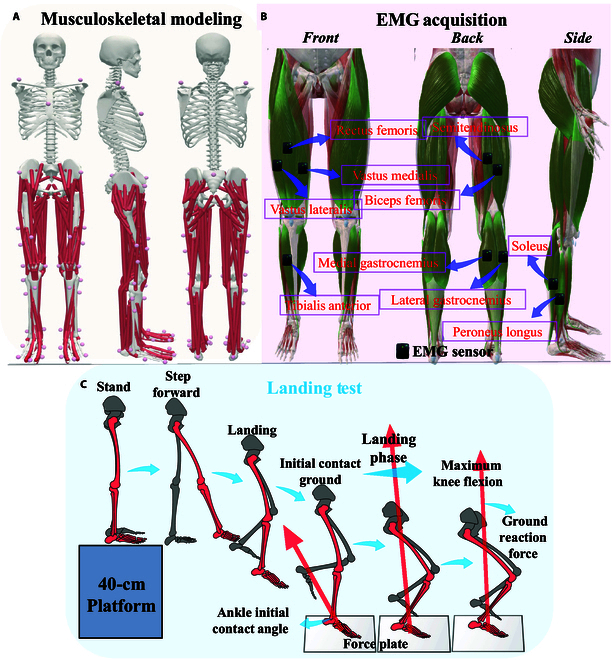
Overview of the data collection and the ACL model. (A) Illustration of the reflective mark’s position about the updated musculoskeletal model. (B) Illustration of the surface EMG test’s position on lower limbs. (C) Illustration of the process of SL biomechanics test.

As shown in Fig. 1, 38 reflective markers were placed on the body to track movement (Fig. 1A), and 10 wireless EMG sensors were used for the surface EMG signal acquisition (Fig. 1B). During EMG signal acquisition, we first treated the skin surface with alcohol cleaning and wiping to ensure that the electrodes could adhere well and record muscle activity accurately [5]. The EMG sensors were then placed jointly by 2 experimental operators to ensure that their electrodes were placed in the middle of the target muscle and aligned with the orientation of the muscle fibers [38]. Besides the use of EMG patches, we used a bandage for secondary fixation of the EMG sensors to ensure that the electrodes would remain stable and in close contact with the muscles. Before starting the formal data acquisition, we also performed signal testing to ensure that the electrodes were properly placed and could accurately record muscle activity. For the adjacent muscles, such as the soleus and lateral gastrocnemius, 2 experimental operators simultaneously observed the EMG signal data during the acquisition in real time. For the observed abnormal fluctuation signal, it is considered an acquisition failure, and the acquisition will be carried out after debugging and checking again.

For these 10 muscles, the corresponding maximal voluntary contraction (MVC) was also collected to calculate the muscle activation [39]. The placement of reflective markers was conducted by the same experimenter for all subjects and checked by a different experimenter. All subjects were required to wear leggings and uniform shoes. After the warm-up (run at their own pace for 10 min), the subjects were familiarized with the experimental procedures. Once the reflective markers and EMG sensors were attached, subjects were required to stand in a standard anatomical position on the force plate with their feet open, shoulder width apart, and arms open to 45° diagonally downward, and they were visually ahead and kept motionless until the experimenter completed static data collection.

The whole test process of the SL biomechanics test is outlined in Fig. 1C. A 40-cm-high jump platform was placed directly in front of the force plate, and the subject stood on the jump platform with his hands on his hips. After hearing the “begin” signal from the experimenter, the subject moves the dominant leg forward, and leans forward to fall vertically from the jump platform at no initial speed synchronously. All subjects were asked to try their best to pause briefly for half a second after leaning forward to ensure no initial velocity while falling from the platform. We determined it in the Vicon Nexus software by observing the real-time spatial displacement changes of the medial and lateral ankle reflective markers. When the reflective marker is observed to have no obvious displacement change for 100 time points (0.5 s), the landing test is considered valid. Subjects were instructed to land with their dominant leg as close to the center of the force plate as possible, and then land on one leg for support and balance. A successful experiment was defined as the subject's ability to balance on the dominant leg for 3 s without any tendency to fall. Five successful landing trial datasets were collected for each subject. The subjects rested for at least 30 s between each session in the landing test.

### Data initial processing and collection

The data of the landing phase from the initial contact force point (VGRF > 10 N) to the maximum knee flexion was selected for analysis [3,6]. Firstly, the data were processed in Vicon Nexus software: (a) name the captured reflective markers; (b) fix the missing reflective markers; and (c) delete the redundant and wrong reflective markers. After that, the exported C3D files from Vicon Nexus were imported into Visual 3D 6.7.3 (C-Motion Inc., Germantown, US) software for the further modeling process. The joint kinematics and joint kinetics were calculated by using the built-in algorithms in Visual 3D. In the specific modeling, we determined the anatomical bone position of the subject through the reflective marker points [1]. By combining the input height and weight, we were able to determine the specific rigid segment ratio for each subject. In the calculation process, lower limb joint angles and angular velocities were calculated based on the collected data of displacement changes of reflective marker points in space over time. In conjunction with the synchronized collected ground reaction force data, an inverse dynamics algorithm then calculated the joint reaction forces and joint moments. The joint power is defined as the product of the joint moment and the joint angular velocity [2].

Based on the calculated most appropriate signal-to-noise ratio, the fourth-order zero-phase lag Butterworth low-pass filters with frequencies of 10 and 20 Hz were used to filter the kinematic and kinetic data [2,40]. Among them, each joint energy dissipation (joint negative/eccentric work) was calculated by the integral of joint power over time [4]. The TED was defined as the sum of ankle, knee, and hip joint energy dissipation. This work only considered joint energy dissipation in the sagittal plane, as the lower limb energy dissipation is mainly concentrated in the sagittal plane during SL [2,4].

The EMG activation results were obtained from the EMG sensors, which were compared with the results obtained by OpenSim to validate the musculoskeletal model [5,41]. First, the raw EMG signals were processed firstly by band-pass filtering with a Butterworth fourth-order filter (frequency range of 10 to 400 Hz). Then, the full-wave rectification was conducted. Based on the determined most appropriate signal-to-noise ratio, the low-pass filter with a 6-Hz cutoff frequency was used [5,42]. EMG signal was normalized by dividing the maximum EMG amplitude of maximal voluntary contraction (MVC) by the maximum root mean square amplitude to obtain the normalized signal *e_i_*(*t*). We used a recursive non-linear model (second-order differential equation) to solve the muscle activation *a_i_*(*t*) by the obtained normalized signal *e_i_*(*t*) [38,41,43]. First, we used the normalized signal *e_i_*(*t*) to solve the neural activation:

$$u_{i}\left(t\right)=\alpha e_{i}\left(t-d\right)-\beta_{1}u_{i}\left(t-1\right)-\beta_{2}u_{i}\left(t-2\right)$$ (1)

where *t* represents the activation time point, and *u_i_*(*t*) is related to the previous activations: *u_i_*(*t* − 1), *u_i_*(*t* − 2). The electromechanical delay *d* is set to be 10 ms, and *α*, *β*_1_ = *C*_1_ + *C*_2_, *β*_2_ = *C*_1_ × *C*_2_ (|*C*_1_| < 1, |*C*_2_| < 1) are the coefficients defining the second-order dynamics [38]. These parameters map *e_i_*(*t*) to *u_i_*(*t*), which is the key to forming a stable equation, and must satisfy the conditions *α* = 1 + *β*_1_ + *β*_2_. Then, the muscle activation *a_i_*(*t*) was solved by the non-linear model [38,43]:

$$a_{i}\left(t\right)=\frac{e^{A_{i}u_{i}\left(t\right)}-1}{e^{A_{i}}-1}$$ (2)

where *A_i_* is set to 1.5, which is the nonlinear shape coefficient, and represents the degree of nonlinearity of the neural activation *u_i_*(*t*) and muscle activation *a_i_*(*t*) [38,41]. All data were imported into MATLAB (Visual R2022a, MathWorks, USA) to expand into 101 data point curves (0% to 100% landing phase) by self-written MATLAB scripts.

### Nonlinear ACL model creation and property setting

**Fig. 2.**
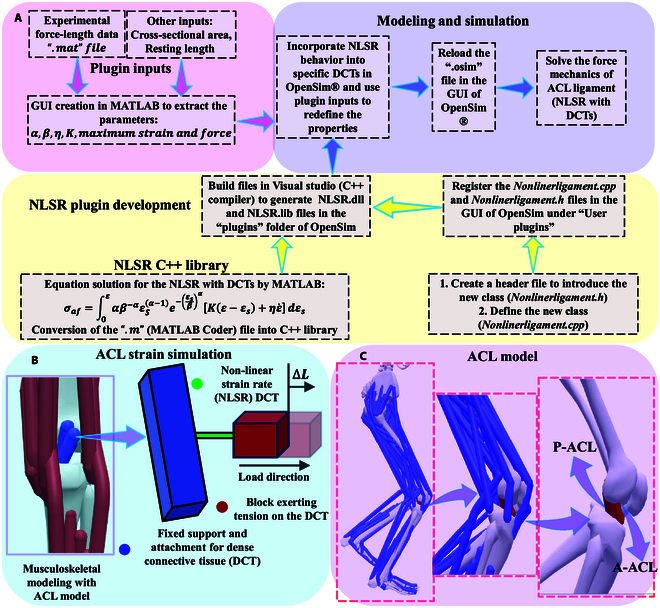
Illustration of the ACL model construction and property setting. (A) Flow diagram of the NLSR plugin creation and DCT model simulation. (B) Illustration of the ACL strain simulation (DCTs). (C) Illustration of the ACL model that was constructed in OpenSim and MATLAB.

When evaluating the mechanism of ACL injury, the traditional ACL model primarily sets ligaments as having linear force–length characteristics and considers them independent of strain rate [30–32]. However, as DCT, ligaments are characterized by short-term viscoelastic strain, which affects their force–length characteristics [31,33]. In order to more realistically revert and simulate the ACL injury mechanics, this study developed a knee musculoskeletal model that reverts the ACL ligament to a nonlinear short-term viscoelastic mechanical mechanism (strain rate-dependent) generated by the DCT as a function of strain. By implementing the structural constitutive model, this study calculated the compressive stress on the ligament at different strains [33,34]. As presented in Fig. 2, we developed a graphical user interface (GUI) for OpenSim based on App Designer in MATLAB to assess the ligament material parameters. Meanwhile, ligament elongation and DCT forces were calculated based on the muscle and force analysis tools in the OpenSim analysis toolbox (Fig. 2A). Considering the short-term viscoelastic behavior of ligament DCTs, the current study fits a sixth-order polynomial equation in the GUI to calculate the material parameters [33,34]. The coefficient matrix ${\bar{p}}_{m,n}$ of this sixth-order polynomial equation is represented as follows:

$${\bar{p}}_{m,n}=\frac{\sum_{m=1}^{K}p_{m,n}}{L}\;\left(n=1,2,\ldots m\right)$$ (3)

where *K* is the sample size for this coefficient matrix equation, and *L* is the number of coefficients. The coefficient matrix result is calculated from the coefficient term *p*_*m*, *n*_ for each sample *n*. We created a *C++* library (containing the *equation*. *h* and *equation*. *cpp* files) through the Win64 *C++* compiler to build and simulate the proposed intrinsic model of the ligament structure. In this process, an adaptive product algorithm is used to solve the numerical integration [44]. Build the NNL class in Visual Studio (Microsoft Ltd. 2017) and then create the NLSR.dll and NLSR.lib folders in the OpenSim plugin to register the NLL class into the OpenSim GUI. Based on the constructed structural constitutive model, this work inputs the material parameters and mechanical parameters of the ligament DCT in the GUI to define the properties of the DCT bundle (Fig. 2B). Finally, the nonlinear characteristic strains and internal loading forces of the ligament under different strains were calculated based on the analysis tools in OpenSim.

The ACL length was defined as the resting length *L_r_* based on the state when the knee was in the neutral position, and the extended length of the ACL when the knee was flexed during SL was defined as *L_e_*, and the ligament strain was calculated by the percentage of *L_e_* to *L_r_*:

$$\varepsilon_{s}=\frac{L_{e}-L_{r}}{L_{r}}\;$$ (4)

Among them, there is a “ligament-class” property in OpenSim, which means that there is a defect in that the extension length cannot be calculated during the dynamic simulation of SL [31]. Therefore, in this study, the instantaneous strain rate (ISR) $\dot{\varepsilon }$ was calculated by differentiating the strain with respect to time [33]:

$$\dot{\varepsilon }=\frac{d\varepsilon_{s}}{\mathrm{dt}}\;$$ (5)

Then, by assuming that the mean axial direction of the ACL collagen fibers is parallel to the loading direction of the stress (*F_a_*), the probability density function defining the fiber orientation under the orientation coefficient *m* of the collagen fibers is:

$$R\left(m\right)=\delta \left(m-F_{a}\right)$$ (6)

where *δ* is the Dirac delta function. Constructing computational functions for nominal axial stress based on strain and strain rate [33,34]:

$$\sigma_{\mathrm{af}}=-p\lambda^{-1}+\sigma_{e}\left(\lambda \right)+\sigma_{v}\left(\lambda ,\dot{\lambda }\right)$$ (7)

The boundary conditions are set to no traction, that is, the pressure term *p* is equal to 0 [34]. *λ* represents the ratio of ligament extension length *L_e_* relative to resting length *L_r_*:

$$\lambda =\frac{L_{e}}{L_{r}}$$ (8)

*σ_e_* is the elastic stress generated in the collagen fibers of the ligament:

σeλ=∫1λRλsσe¯λλsdλsσe¯λ=Klnλ (9)

where $\bar{\sigma_{e}}$ is the elastic stress generated in each collagen fiber of the ligament. *R*(*λ_s_*) represents the probability density function of the fiber activity density of the ligament in the stretched state (*λ_s_*). *K* is a constant, taken as 70 MPa, which is used to represent the collagen fiber’s elastic modulus [45]. *σ_v_* is the viscous stress generated in the collagen fibers of the ligament:

σvλ=∫1λRλsσv¯λλs,λ˙λsdλsσv¯λr,λr˙=ηDDtlnλr (10)

where *λ_r_* is the stretch ratio of *λ* and *λ_s_*, and *η* represents the absolute viscosity coefficient, taken as 20 MPa/S [45]. The term $\frac{D}{D_{t}}$ represents the time derivative of matter. Therefore, combining Eqs. 7, 9, and 10, the following equation can be obtained:

$$\sigma_{\mathrm{af}}=\int_{1}^{\lambda }R\left(\lambda_{s}\right)\left\{K\ln\frac{\lambda }{\lambda_{s}}+\eta \frac{D}{D_{t}}\left(\ln\frac{\lambda }{\lambda_{s}}\right)\right\}d\lambda_{s}\;$$ (11)

Based on previous studies [33,34,45,46], distribution functions were used to solve for the uncoiled stretch of collagen fibers to obtain the probability distribution of their unwinding *R*(*λ*):

$$R\left(\lambda \right)=\left(\frac{\mathrm{ln\lambda}-\gamma }{\beta }\right)\frac{\alpha }{\beta }{\left(\frac{\mathrm{ln\lambda}-\gamma }{\beta }\right)}^{\alpha -1}\exp\left(-{\left(\frac{\mathrm{ln\lambda}-\gamma }{\beta }\right)}^{\alpha }\right)\;$$ (12)

where *α*, *β* > 0, are the shape and scale with boundary conditions, respectively. γ is the location parameter, when it converges to zero based on the Weibull model [34] *λ_s_* = *e^ε_s_^*. Thus, the final calculation function of the nominal axial stresses developed based on the DCT is

$$\sigma_{\mathrm{af}}=\int_{0}^{\varepsilon }\alpha \beta^{-\alpha }\varepsilon_{S}^{\left(\alpha -1\right)}e^{-{\left(\frac{\varepsilon_{s}}{\beta }\right)}^{\alpha }}\left[K\left(\varepsilon -\varepsilon_{s}\right)+\eta \dot{\varepsilon }\right]d\varepsilon_{s}$$ (13)

where *α* = 4.5 and *β* = 0.3 are the shape and scale factors of the probability distribution function, respectively, for the sequential straightening of collagen fibers under stress loading in the 2-parameter Weibull model [33,34,45].

Based on the mechanical and material properties of ligaments, the ligament fiber bundle model is defined as a nonlinear viscoelastic unit [5,33]. When the ligament is in a resting or slack state, that is, the strain is equal to or less than zero, then the compressive stress within the ligament is zero. When the ligament is in tension, the strain is greater than 0, the internal ligament loading force is *F_af_*:

$$F_{\mathrm{af}}=\sigma_{\mathrm{af}}A$$ (14)

where *A* is the average physiologic cross-sectional area of the DCTs. Based on our previous study, the anteromedial ACL (A-ACL) and posterolateral ACL (P-ACL) were modeled using the DCTs (Fig. 2C), and a tunnel connecting the femur and tibia at both ends [5,45]. The ACL was attached to the medial front of the tibial intercondylar eminence and extended to the medial side of the lateral condyle of the femur. For the A-ACL, the resting length is set to 30 mm, the cross-sectional area is set to 20.7 mm^2^, and the max isometric force is set to 1,500 N. For the P-ACL, the resting length is set to 23.36 mm, the cross-sectional area is set to 19.3 mm^2^, and the max isometric force is set to 1,600 N. The final calculated total ACL force was determined as the combination of the A-ACL force and the P-ACL force [5].

The lower limbs will dramatically change the acceleration and GRF of each segment during the landing phase, particularly in the early landing stage, which will prevent the optimization process from being convergent. As a result, during the whole modeling and simulation process, many attempts were performed to simulate the landing using the Computed Muscle Control (CMC) and Reduce Residuals Algorithm (RRA) for each segment. The strain of the ACL varies with the knee valgus angle under different flexion conditions. The ACL strain can be seen as a function of knee kinematics according to the simulation results after CMC, which is the function of the muscle optimization process. Therefore, this work sets the ratio of passive ACL strain to flexion-extension and varus-valgus angle to 15% by adjusting the ACL material properties for passive fiber strain at the maximum isometric force [31,47–49]. As a maximum tolerance ratio, 15% is reasonable because the range of 9% to 15% has been shown to cause ACL rupture and microfiber damage [5,49,50]. If a passive ACL strain characteristic is specified as less than 1% in an individual model, additional adjustments are made to the models to ensure that the strain limit can be accurately controlled. For the contact area between ligament and bone (junction of ligament together with bones), the maximum strain was limited to 2.5% to 3.0% to ensure that the isometric force could start from the lowest value in the simulation [47]. The length of bony osteoligamentous (tendons) and ligaments (muscles) will be examined during CMC based on the determined ACL strain when the presence of deformation of the entire muscle–tendon unit [5,31].

### Statistical analysis

Data analysis was conducted in SPSS 27.0 (IBM Corporation, NY, USA). Before formal analysis, the Kolmogorov–Smirnov test was performed to determine the normality of the data for each variable. All data obeyed a normal distribution. For the relationship between the ankle motion patterns (AICA and AROM) and lower limb injury risk (peak VGRF [PVGRF], total energy dissipation [TED], peak ankle dorsiflexion moment [PADM], peak knee flexion moment [PKFM], peak hip flexion moment [PHFM], peak ACL force [PAF], peak ankle inversion angle [PAIA], and peak ankle inversion moment [PAIM]), the Pearson correlation and linear regression analysis were conducted to explore them (significant level *P* < 0.05). The value of Pearson correlation coefficient *r* ranges from −1 to 1. *r* > 0, *r* < 0, and |*r*| = 1 represent the positive, negative, and no correlation, respectively. |*r*| = 1 indicates a perfectly linear correlation.

Meanwhile, the PAF and PAIA were standardized (0 to 1) to enable PAF and PAIA to be compared on the same frame of reference. This allows an effective assessment of the interaction between ACL injury and LAS risk. The corresponding maximum PAF and PAIA were estimated as the maximum likelihood of injury risk occurring, and the minimum PAF and PAIA were estimated as the minimum likelihood of injury risk occurring [5]. Therefore, the injury risk calculation formula is as follows:

$$R_{\text{injury risk}}=\frac{\mathrm{PAF}/{\text{PAIA}}_{\text{current value}}-\mathrm{PAF}/{\text{PAIA}}_{\text{minimum value}}}{\mathrm{PAF}/{\text{PAIA}}_{\text{maximum value}}-\mathrm{PAF}/{\text{PAIA}}_{\text{minimum value}}}$$

where *PAF*/*PAIA_current value_*, *PAF*/*PAIA_maximum value_*, and *PAF*/*PAIA_minimum value_* correspond to the current, maximum, and minimum values of PAF/PAIA, respectively.

## Results

### Muscle activation and raw waveform data during the landing phase

**Fig. 3.**
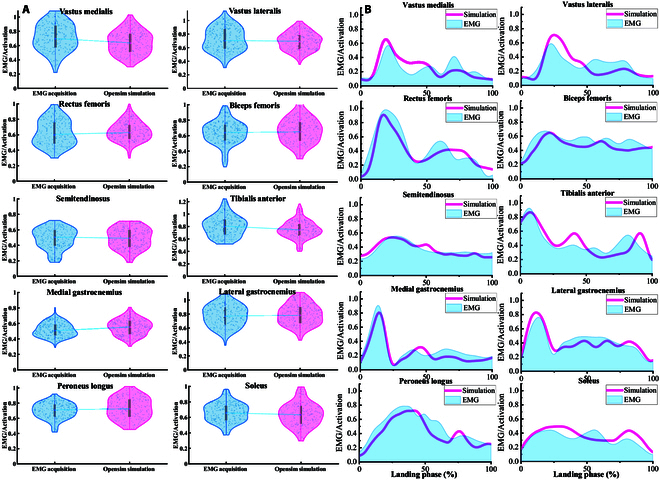
Illustration of the EMG/Activation of selected muscles. (A) Comparative results of muscle activation degrees for all subjects. (B) Comparative results of time-dependent muscle activation for a typical subject.

**Fig. 4.**
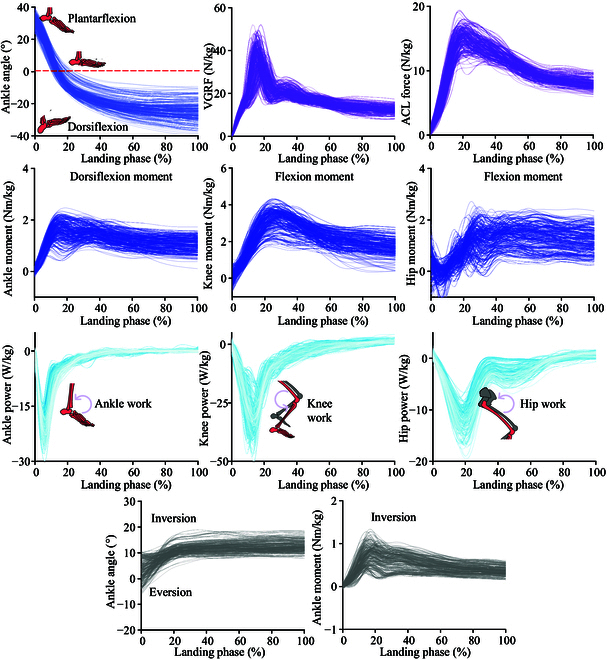
Visualization of the raw data waveform of the relevant parameters during the landing phase.

Figure 3 presents the results of the comparison between the muscle activation results collected by the EMG sensor and the OpenSim musculoskeletal model simulation results. In both cases, the activation levels of the 10 selected muscles remained essentially the same, suggesting that the model constructed in this study has a high degree of reliability [31,39]. The raw data waveform of the relevant parameters during the landing phase is shown in Fig. 4.

### Relationship between the overall injury risk of lower limb and ankle motion patterns

**Fig. 5.**
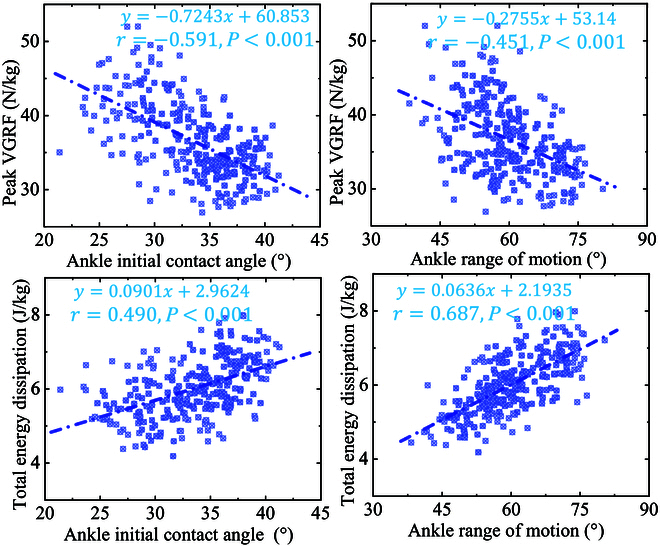
Visualization of the linear relationship and scatter distribution between PVGRF and TED, and AICA and AROM.

**Fig. 6.**
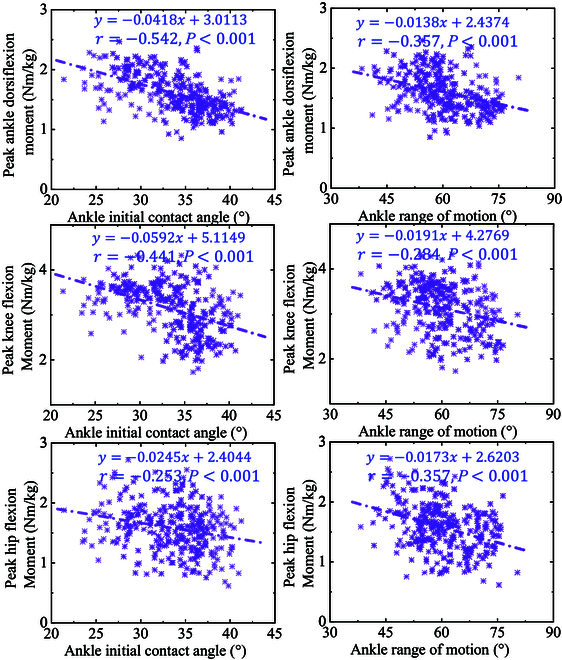
Visualization of the linear relationship and scatter distribution between peak ankle, knee, and hip sagittal moment, and AICA and AROM.

There is a correlation between ankle movement patterns and overall injury risk (Table and Fig. 5 and 6). The results of the AICA and AROM are 33.47±5.16° and 59.99±8.35°, respectively (Table 1). The linear relationship and scatter distribution between PVGRF and TED, and AICA and AROM are shown in Fig. 5, and the detailed values are provided in Table 1. With the increase of AICA during SL, the PVGRF shows a downward trend (*r* = −0.591, *P* < 0.001). With the increase of AROM during landing, the PVGRF shows a downward trend (*r* = −0.451, *P* < 0.001). With the increase of AICA during landing, the TED shows an upward trend (*r* = 0.490, *P* < 0.001). With the increase of AROM during landing, the TED shows an upward trend (*r* = 0.687, *P* < 0.001).

**Table.** Detailed results of the Pearson correlation coefficients between ankle motion patterns and lower limb variables

Variables	Mean ± SD	Ankle initial contact angle (33.47° ± 5.16°)			Ankle range of motion (59.99° ± 8.35°)		
		*r*	*R* ^2^	*P*	*r*	*R* ^2^	*p*
Peak VGRF (N/kg)	36.61 ± 5.10	−0.591	0.349	<0.001	−0.451	0.203	<0.001
Total energy dissipation (J/kg)	6.01 ± 0.77	0.490	0.241	<0.001	0.687	0.472	<0.001
Ankle sagittal moment (Nm/kg)	1.61 ± 0.32	−0.542	0.294	<0.001	−0.357	0.128	<0.001
Knee sagittal moment (Nm/kg)	3.13 ± 0.56	−0.441	0.194	<0.001	−0.284	0.081	<0.001
Hip sagittal moment (Nm/kg)	1.58 ± 0.40	−0.253	0.064	<0.001	−0.357	0.128	<0.001
Ankle inversion angle (°)	14.19 ± 2.74	0.502	0.253	<0.001	0.600	0.360	<0.001
Ankle inversion moment (N/kg)	0.75 ± 0.23	−0.268	0.072	<0.001	−0.138	0.019	0.168
Peak ACL force (N/kg)	14.72 ± 1.68	−0.554	0.307	<0.001	−0.332	0.110	<0.001

The linear relationship and scatter distribution between peak ankle, knee, and hip sagittal moment, and AICA and AROM is shown in Fig. 6. With the increase of AICA during landing, the peak ankle (*r* = −0.542), knee (*r* = −0.441), and hip (*r* = −0.253) sagittal moment all show a downward trend (Table 1). With the increase of AROM during landing, the peak ankle (*r* = −0.357), knee (*r* = −0.284), and hip (*r* = −0.357) sagittal moment all show a downward trend (Table 1).

### Relationship between the ACL injury risk and ankle motion patterns

**Fig. 7.**
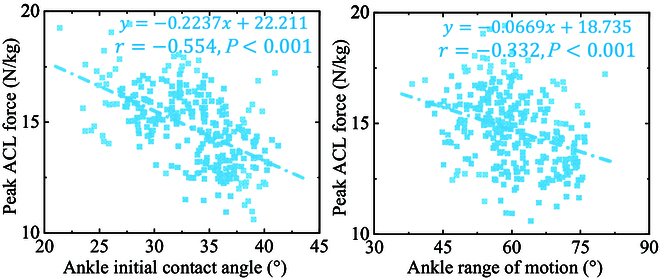
Visualization of the linear relationship and scatter distribution between peak ACL force and AICA and AROM.

AICA and AROM are negatively correlated with ACL injury risk (Fig. 7). With the increase of AICA during landing, the PAF shows a downward trend (*r* = −0.554, *P* < 0.001). With the increase of AROM during landing, the PAF shows a downward trend (*r* = −0.332, *P* < 0.001) (Table 1).

### Relationship between the LAS risk and ankle motion patterns

**Fig. 8.**
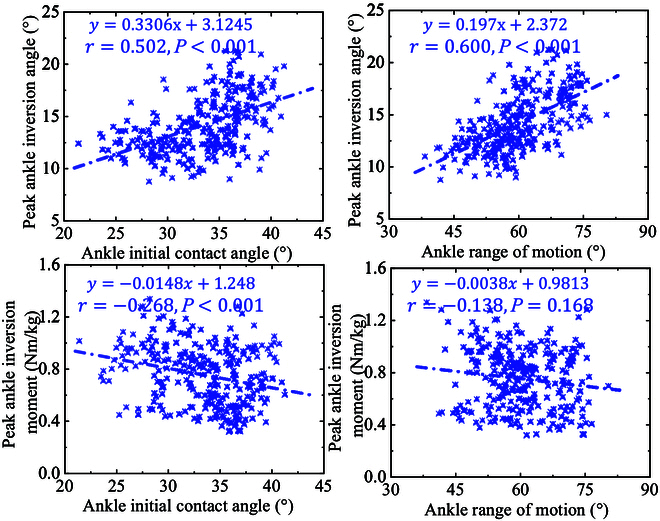
Visualization of the linear relationship and scatter distribution between peak ankle inversion angle and moment, and AICA and AROM.

AICA and AROM are positively correlated with LAS risk (Fig. 8). For the PAIA, it shows an upward trend with the increase of AICA (*r* = 0.502, *P* < 0.001) and AROM (*r* = 0.600, *P* < 0.001) (Table 1). For the PAIM, it shows a downward trend with the increase of AICA (*r* = −0.268, *P* < 0.001) and AROM (*r* = −0.138, *P* = 0.168) (Table 1).

### Interaction between LAS risk and ACL injury risk

**Fig. 9.**
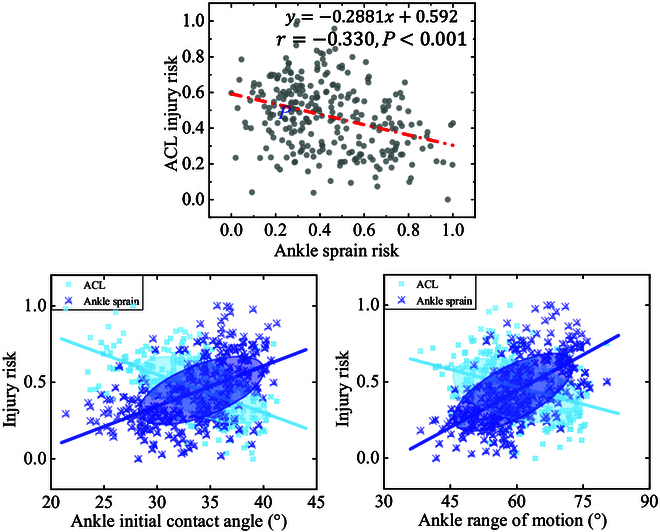
Visualization of the interaction between the risk of ACL injury and ankle sprain, AICA, and AROM. The closer the injury risk is to 1, the greater the probability of the corresponding injury.

As shown in Fig. 9, the LAS risk is negatively correlated with ACL injury risk (*r* = −0.330, *P* < 0.001). For the AICA, the intersection of PAF and PAIA occurs at 34.09°, and the approximate range of balanced LAS and ACL injury risk may be 30° to 40°. For the AROM, the intersection of PAF and PAIA occurs at 61.18°, and the approximate range of balanced LAS and ACL injury risk may be 50° to 70°.

## Discussion

This work aims to explore an optimized landing strategy to reduce the injury risk of the lower limb. Specifically, we explored the relationship between ankle joint motion patterns and lower limb injury risk during SL, as well as investigated how to balance the association between the LAS and ACL injury risk under different ankle motion patterns. We hypothesized that the overall injury risk of the lower limb, particularly ACL injury, can be minimized by appropriately increasing AICA and AROM during SL, but this increases the LAS risk. The current findings partially support the hypothesis that the larger AICA and AROM exhibit smaller PVGRF and PAF, as well as larger TED and PAIA. In addition, based on the interaction between PAIA and PAF, we confirmed the range of the AICA and AROM that can balance the LAS and ACL injury risk.

During landing, the subject usually adopts ankle plantarflexion, and then transitions to dorsiflexion until reaching the steady state. In this study, the maximum ankle plantarflexion angle AICA was mainly distributed in the range of 20° to 40° (33.47° ± 5.16°) based on the ankle anatomical position in normal standing at 0°. With the increase of the AICA and AROM, PVGRF exhibited a negative correlation with them, and the correlation coefficient of AICA (*r* = −0.591) was higher than that of AROM (*r* = −0.451). This indicates that using a larger plantarflexion angle is an effective way to reduce lower limb impact loads during SL. It also supports previous findings that greater AICA and AROM reduced PVGRF and increased time to PVGRF [7,29,51]. Overall, a larger AICA during SL allows the body to use the plantar flexors to attenuate and cushion the impact load to a greater extent, with the accompanying larger AROM also dissipating more load impact [1,7,51].

In addition, the joint power is shown in Fig. 4, the negative joint power represents that the joint extensor muscles dissipate the impact by producing eccentric work, and the higher negative joint power means a greater eccentric distance of the joint extensor muscle [2,4]. By integrating and calculating the joint power and obtaining TED, we found that TED presented a relatively obvious positive relation with AICA (*r* = 0.490) and AROM (*r* = 0.687) (Fig. 5). Firstly, it is undeniable that the increase of AICA drives the increase of AROM to a certain extent, which also leads to an increased demand for active muscle contraction of the distal joints, with a consequent increase in TED of the lower limb joints. The increase in TED suggests that the lower limb is well cushioned against impact loads, and then the load is reduced based on the passive joint structures, thus reducing the lower limb injury risk [2,12]. Therefore, the current results demonstrate that changes in ankle motion patterns during SL can influence the degree of impact loading on the lower limb, as well as alter the joint ability to dissipate energy during transfer.

To further radiate the specific effect on each joint, the relationships between AICA, AROM, peak ankle sagittal moment, peak knee sagittal moment, and peak hip sagittal moment were explored. The results showed that both AICA and AROM exhibited negative correlations with each joint moment, with a stronger correlation between AICA and PADM (*r* = −0.542) and PKFM (*r* = −0.441). The magnitude of the sagittal joint moment is directly related to the joint injury risk during landing [1]. As shown in Fig. 6, we can find that the increase of AICA and AROM may reduce the injury risk of each joint to a certain extent, which also confirms the conclusion that has been derived above.

The reduction of AICA could raise the energy dissipation demand of the proximal joint, which would result in an increased ACL injury risk. Previous studies have also speculated that larger body anteverted angle and ankle plantarflexion in contact with the ground during SL may show better lower limb cushioning ability with less ACL injury [29,51]. According to Lee and Shin [52], there is a negative correlation between the AICA and both the peak knee valgus moment (*r* =  −0.5) and the total of the internal rotation moments and peak knee valgus (*r* =  −0.58). However, there are no relevant studies that directly demonstrate this from the dynamic loading of ACL itself, which often has a certain bias risk. Based on this, this study developed and constructed the ACL model in the OpenSim for musculoskeletal modeling simulations to calculate ACL dynamic loading forces during landing, and explored the relationship between ACL injury risk and ankle motion pattern.

As shown in Fig. 8, the present work found that both AICA and AROM showed a negative correlation with PAF, with AICA (*r* = −0.554) showing a higher correlation than AROM (*r* = −0.332). The results suggested that increasing the AROM and AIC during SL would increase joint energy dissipation and lower the impact loads on the joints, thereby lowering the injury risk of the lower limb, including ACL injuries. Both AICA and AROM can be adjusted by autonomous awareness during SL, and larger AICA is often accompanied by larger AROM. As a result, we propose that the individual can actively and consciously increase the AICA and AROM during SL to increase the TED, thus reducing lower limb injury risk. The muscles and tissues surrounding the ankle joint must be very strong in order to consciously increase AICA and AROM [5]. When dispersing impact stresses during SL, the ankle joint mostly depends on the muscle and tendon units surrounding it [1,5,29]. In the absence of strengthened tissues and muscles surrounding the ankle to accommodate the increased AIC and AROM, the LAS risk increases during energy impact dissipation [5].

In this study, AICA and AROM showed a strong positive correlation with PAIA, and a weak negative correlation with PAIM (Fig. 8). The increased PAIA is believed to increase the LAS risk, which can further cause ankle instability and thus affect lower limb function [6,9,53]. Hence, it is essential to determine a feasible ankle motion pattern to balance the LAS and ACL injury. As shown in Fig. 9, the results indicated that the LAS risk is negatively correlated with ACL injury risk (*r* = −0.330, *P* < 0.001). Based on the determined intersection points, we found that 30° to 40° of AICA and 50° to 70° of AROM were the more appropriate range to balance the injury risk between them. This range can be referenced by individuals during SL, but it should also be adjusted according to the person’s ankle dorsiflexion ability and the level of muscle function around the ankle joint. In addition, it is also necessary to strengthen the training of the muscles, the medial and lateral tissues, and ligaments around the ankle joint, so as to increase the AICA and AROM to reduce lower limb injury risk while avoiding ankle joint injury.

Based on the actual situation in constructing ACL models, we summarize the following optimization directions, hoping to make a breakthrough in future research. Throughout the simulation calculations, the model only considers the effect of knee motion on the ACL, but does not consider that as the knee joint movement changes, the attached ACL ligament will in turn affect the DOF of the knee joint. However, in the real human body, the knee DOF and ACL interact with each other. The changes in the knee joint can cause ACL strain, which is transmitted to the knee joint structure, and then affects the range of motion and stability of the knee joint [54]. Due to the complexity of the real human body environment, it is difficult for musculoskeletal modeling and simulation to completely restore the real state [55]. In our future research, we hope to further optimize and develop a more accurate and realistic musculoskeletal model by considering the interaction between various structures.

This study is a relatively short-term landing action simulation, so the ACL mainly exhibits a tensile strain state. This situation only needs to consider the viscoelastic properties of ACL under increasing tensile strain, without considering the viscoelastic response of ACL between stretching and shortening [1,5]. Meanwhile, considering that setting ACL as a long-term viscoelastic property may increase model complexity and computational cost, the current study sets ACL as a nonlinear short-term viscoelastic property. Future research can focus on further deepening musculoskeletal modeling to develop an ACL model with long-term viscoelastic properties.

In this work, the parameters of the ACL, such as origin, insertion point, length, and cross-sectional area of the ligament, were mainly based on data from previously published studies. In view of personalized differences, future research can consider combining medical imaging data (magnetic resonance imaging and computed tomography) to construct a subject-specific ACL model of the knee joint. This can make the musculoskeletal modeling simulation more closely match the real situation of the human body, and improve the accuracy and reliability of the simulation results [56,57]. On the other hand, researchers may also consider combining machine learning and data-driven approaches to optimize the model parameters with the help of a large amount of experimental and simulation data [5]. This can improve the accuracy and generalization ability of ACL force prediction models.

The current work also has the following 2 limitations and outlooks. First, only the male subjects were studied in this study, and it is not clear whether the movement laws explored based on the results are still applicable to females. The next work should consider exploring the inherent laws in the SL patterns of female subjects. In addition, when assessing the ankle injury risk, we used only the indicator of ankle inversion angle. Although this indicator is largely indicative of the degree of LAS risk, future research should focus on more detailed indicators given the complexity of the medial and lateral ligaments around the ankle [1,58].

In conclusion, the current results revealed that the appropriate increases in AICA and AROM may reduce the lower limb injury risk, particularly ACL injuries, but this may increase the LAS risk. AICA in the approximate range of 30° to 40° and AROM in the approximate range of 50° to 70° are likely to balance the association between the LAS and ACL injury risk. It is essential to strengthen the training of the muscles, the medial and lateral tissues, and ligaments around the ankle. This can reduce lower limb injury risk while avoiding ankle joint injury when AICA and AROM are increased. This study has the potential to offer novel perspectives on the optimized application of landing strategies, thus giving crucial theoretical backing for decreasing the injury risk of the lower limb during SL.

## Data Availability

All data relevant to the current study are included in the article; further inquiries can be directed to the corresponding author.
